# Influence of Snowmelt Timing on the Diet Quality of Pyrenean Rock Ptarmigan (*Lagopus muta pyrenaica*): Implications for Reproductive Success

**DOI:** 10.1371/journal.pone.0148632

**Published:** 2016-02-05

**Authors:** Ricardo García-González, Arantza Aldezabal, Nere Amaia Laskurain, Antoni Margalida, Claude Novoa

**Affiliations:** 1 Instituto Pirenaico de Ecología (CSIC), Jaca, Spain; 2 Plant Biology and Ecology Department, Basque Country University, Bilbao, Spain; 3 Department of Animal Production, Division of Wildlife, Faculty of Life Sciences and Engineering, University of Lleida, Lleida, Spain; 4 Division of Conservation Biology, Institute of Ecology and Evolution, University of Bern, Bern, Switzerland; 5 Office National de la Chasse et de la Faune Sauvage, Prades, France; Université de Sherbrooke, CANADA

## Abstract

The Pyrenean rock ptarmigan (*Lagopus muta pyrenaica*) is the southernmost subspecies of the species in Europe and is considered threatened as a consequence of changes in landscape, human pressure, climate change, and low genetic diversity. Previous studies have shown a relationship between the date of snowmelt and reproductive success in the Pyrenean ptarmigan. It is well established that birds laying early in the breeding season have higher reproductive success, but the specific mechanism for this relationship is debated. We present an explicative model of the relationship between snowmelt date and breeding success mediated by food quality for grouse in alpine environments. From microhistological analyses of 121 faecal samples collected during three years in the Canigou Massif (Eastern Pyrenees), and the assessment of the chemical composition of the main dietary components, we estimated the potential quality of individual diets. Potential dietary quality was correlated with free-urate faecal N, a proxy of the digestible protein content ingested by ptarmigan, and both were correlated with phenological stage of consumed plants, which in turn depends on snowmelt date. Our findings suggest that the average snowmelt date is subject to a strong interannual variability influencing laying date. In years of early snowmelt, hens benefit from a longer period of high quality food resources potentially leading to a higher breeding success. On the contrary, in years of late snowmelt, hens begin their breeding period in poorer nutrient condition because the peaks of protein content of their main food items are delayed with respect to laying date, hence reducing breeding performance. We discuss the possible mismatch between breeding and snowmelt timing.

## Introduction

Optimal selection of breeding onset can be important for reproductive success in birds [1,2]. Earlier laying is often associated with larger clutch sizes and greater production of young [3,4]. Conversely, a delay in laying may result in a reduction of nestling growth and survival of fledged young in migratory [5] and sedentary birds [6]. In arctic-alpine birds, a delay in breeding season caused by a late snowmelt or adverse climatic conditions in spring normally results in lower reproductive success [7–11]. On the contrary, earlier snowmelt facilitates the advancement of laying dates increasing breeding success [11–13] and population growth [14]. In this sense, the reproductive success of Pyrenean rock ptarmigan (*Lagopus muta pyrenaica*) was positively associated with the early appearance of snow-free ground [15].

The Pyrenees are home to a small, southern population of rock ptarmigan (<1000 individuals [16]), considered threatened as a consequence of changes in landscape, habitat fragmentation, human pressure, climate change, and low genetic diversity [17]. This small and isolated population may be more prone to extinction due to demographic and environmental stochasticity [18,19] and global change [20], with juvenile survival being a highly influential parameter in ptarmigan populations [21]. Thus, given that data about their ecology in the Pyrenees is relatively scarce [15], any information provided could be important from a conservation point of view, to improve management and conservation actions based on evidence [22].

Clutch timing depends on intrinsic (age, body condition, endogenous rhythms) and extrinsic (photoperiod, climatic conditions, food availability) factors [11]. Some authors suggest that an increase in temperature exerts a direct effect on the onset of reproduction [23]. Other authors have suggested that, the direct relationship between reproductive success and spring temperature is mediated by food quality available to birds before, and during the breeding season [24–26]. Several factors can affect reproductive success [27], but climatic conditions (and particularly the timing of snowmelt) are one of the main factors determining reproductive output in high mountain environments [6,12,13, 28].

We hypothesised that the availability and quality of diet in spring is associated with the timing of snowmelt conditioning beyond the reproductive success of Pyrenean rock ptarmigan. Late snowmelt during spring reduces the availability of food resources for ptarmigan hens at a time when they must also successively cope with moulting, laying and incubation. Furthermore, adverse climatic conditions during the hatching period can negatively affect chick survival which, in their first weeks, require a quantitatively and qualitatively adequate food supply [6,29].

Relying on previous studies of relationships between climate and reproduction, and a detailed description of food habits of a rock ptarmigan population in the Pyrenees [15,30], we present a possible mechanism to explain the mediating role of food quality in the reproductive success of the Pyrenean rock ptarmigan. Specifically, we aim to show that nitrogen content of woody plants (the main food component of grouse diet at the beginning of the growing season) varies depending on the phenological stage (or phenophase), which in turn is closely linked to snowmelt date. Secondly, we model the relationship between the nutritional quality of the ptarmigan diets (expressed as digestible protein content) and the snowmelt date, and demonstrate that diet quality is more dependent on the phenological stage of food components than their floristic composition. Finally, with the influence of the climatic event (snowmelt) on diet quality during the breeding period established, we propose an explanatory model by which the inter-annual variations in the timing of snowmelt in alpine environments may affect the breeding success of rock ptarmigan.

## Materials and Methods

### Ethics statement

The study was conducted in full compliance with France and Spanish laws and regulation, including the licence of Office national de la Chasse et de la Faune Sauvage (ONCFS) and Ordesa y Monte Perdido National Park (PNOMP) to sampling Pyrenean rock ptarmigan faeces and plant species forming part of their diet. Radio-tracking monitoring of ptarmigans was not a part of this particular study, rather than the location of bird for droppings collection in the field, confirming that the field studies related with this study did not involve the management endangered or protected species. The radio monitoring was part of a larger study on demography and habitat use of Pyrenean ptarmigans conducted by the ONCFS, for which all permits and ethical and legal requirements were required and enforced. According to instructions of ONCFS (France) and Ethics Committee of CSIC (Spain), when no animal experiments have been done no other licences were needed.

### Study area

The study was conducted on the Canigou Massif (42°31' N, 2°29' E), which represents the eastern limit of the distribution of rock ptarmigan in the French Pyrenees and the southern limit in Western Europe. The breeding habitat of the species is mainly located on steep slopes and plateaus, at altitudes ranging from 2200 m to 2700 m. At the lowest elevations, the vegetation is dominated by open woodlands of Mountain Pine *Pinus uncinata* associated with ericaceous shrublands (*Rhododendron ferrugineum*, *Vaccinium myrtillus*, *V*. *uliginosum*, *Empetrum nigrum*). The upper parts of the habitat are characterised by gneiss screes mixed with sparse alpine vegetation (*Dryas octopetala*, *Salix herbacea*, *Silene acaulis*, *Saxifraga* spp.) [31]. At 2150 m, mean annual temperature is 4.6°C and mean annual precipitation exceeds 1400 mm. The seasonal distribution of rain shows a high interannual variability, but summer is generally the driest season.

The rock ptarmigan in the Pyrenees breeds in alpine habitats at an average elevation of about 2400 m [32]. At those altitudes, the plant vegetative season lasts on average from mid-June to early September [33], but it is only during plant growing period (June—early July) that protein and nutrient content is higher than in the rest of vegetative period in alpine plants [34]. This relatively short period also represents the average breeding-onset window for Pyrenean rock ptarmigan [15].

### Diet determination

Fresh droppings were collected during monitoring and population surveys throughout the year in an area of 50 km^2^ during three breeding periods (2002 to 2004). In most cases, birds were first located, sex and age class were identified, and then faecal samples were collected. Faecal samples of young were identified by their size. Seasonally, 81 samples (67%) were collected during the spring/summer post-melting period, when the main diet variation took place, and 40 samples (33%) were collected in the autumn/winter period [30]. A total of 121 droppings were collected and analysed by means of microhistological analysis to determine diet composition [35]. This method has been successfully used for investigating the diet of herbivorous birds (Waterfowl: [36]; *Branta bernicla*: [37]; *Perdix perdix*: [38]; *Tetrao urogallus*: [39]). Although we analysed faecal samples separately we did not consider sex and age class categories when analysing diet quality results. This is a study at the population level and we assume diet quality of all sex and age classes (females, males and young) contributed in a less or more degree to the reproductive success of the population. On the other hand, sex/age factor seems to explain only a small proportion of diet variation in this and other rock ptarmigan populations [8,30].

Diet content was ascertained by microscopically identifying leaf, seed, fruit, woody elements and arthropod fragments in the prepared droppings. Usually, only the leaf epidermis has recognisable characters useful in identifying the microscopic fragments at the species or genus level. Flower, seed, fruit or woody elements can normally only be identified at higher taxonomic levels [40]. A plant epidermal collection of the possible grouse food species in the study area was previously assembled. An average of 168 (range 100–364) identifications per individual faecal sample was achieved. Relative frequencies of each dietary item in each faecal sample were calculated.

### Potential nutritional value of diet

Once the main dietary plant species were identified, we collected samples of them in the field to measure their nutritional value. Samplings were carried out at two localities of similar habitat separated by 200 km: the Canigou Massif at 2250 m (France, Eastern Pyrenees 42° 30’N, 2° 27’E) and Calcilarruego at 2000 m (Spain, Central Pyrenees, 42° 38’ N, 0° 3’ W). Sampled species were: *Rhododendron ferrugineum* (Rho fer in graphics and tables), *Salix pyrenaica* and *S*. *herbacea* (hereafter referred as *Salix* spp.), *Vaccinium myrtillus*, *V*. *uliginosum* (referred as *Vaccinium* spp.), *Calluna vulgaris*, *Dryas octopetala*, *Empetrum nigrum* and *Loiseleuria procumbens*. For *Rhododendron ferrugineum*, buds, old leaves, new leaves, flowers and fruits were clipped separately. For the rest of the species, only old and new leaves (depending on phenology) were taken together with their twigs and buds. Twigs thicker than 2 mm were rejected. A total of 545 samplings were carried out in 2005 at 10-day intervals during the plant growing period, i.e. from late May to early July. In the Spanish locality, samplings were prolonged until autumn to obtain a more extended seasonal variation of chemical composition. At each sampling date, five samples (on average) of each item were clipped in different sampling plots depending on species presence. In each sampling plot plant parts were collected by hand or scissors within a radius of nearly 10 m to obtain a minimum of 30 grams of fresh material. Average phenological stage of each species was also noted during plant samplings.

In the laboratory, samples were cleaned, dried at 60°C and milled to a size of 1 mm. Nitrogen (N) content was determined by means of the Kjeldahl method and expressed as a percentage of dry matter (% DM). N content was used as a proxy of crude protein content and diet quality [41]. Protein content is one of the fundamental components of bird nutrition [42,43], and some authors suggest that it is even more important than metabolisable energy when the ptarmigan selects food during the winter [44,45] or breeding period [46].

Potential N content of each individual diet was estimated from the abundance of each food item in faecal samples (microhistological analysis) multiplied by their N content following the expression, *N*_*d*_ = *ΣX*_i_*N_*x*_ where *N*_*d*_ is the potential *N* content of a faecal sample, *X*_*i*_ the proportion of food item *i* in the faecal sample and *N*_*x*_ the *N* content (% of dry matter) of the food item *X* in the respective month or time period (see S1 Table). Nutritive content estimation of mixed or composite diets from the food nutrient content and the proportion of each food item is a common procedure in herbivorous birds [9,47,48]. Potential N content of faecal samples were calculated by month during the breeding season, and for the whole winter period. Items with unknown N content were estimated from bibliographic data. Food items were grouped in simplified categories. N values for plant species and food categories in each time period are shown in the S1 Table.

### Faecal N content

N excretion (total N) is related to N intake [41], but the total N of bird droppings is composed of faecal N and urinary N and they must be separated to obtain a better estimation of the consumed digestible protein than using total N only. [49,50]. We determined free-urate N content of each faecal sample, and used it as a proxy for protein content of individual diets [49]. Faecal samples were added to 10 ml acetic-formalin and recipients were shaken in an ultrasonic bath for one hour to liberate droppings from the urine portion [50]. N content was estimated by the Kjeldahl method before (total N) and after washing (free-urate faecal N), and we considered N content of droppings after washing (in % DM) as a proxy of digestible protein ingested [51] and hence of diet quality. In samples that could not be washed due to small quantities, we estimated free-urate faecal N from the following linear regression: [free-urate faecal N] = 0.518 [total faecal N]– 0.528 (*r*^2^ = 0.705; *n* = 74).

### Climatic data

Snow depth and daily mean temperatures were obtained from two weather stations: Nivose-Cortalets (2150 m) for the Canigou study area and Góriz station (2250 m) for the Calcilarruego study area, located at 12 and 6 km from sampling areas respectively. While snow depth was measured throughout winter and spring by the meteorological Services, we defined the date of snowmelt as the end of the period of continuous snow cover at weather stations [15, 52]. Dates of snowmelt at Canigou, where both droppings and dietary plants were sampled, were: 28/05/2002, 05/05/2003, 08/06/2004 and 16/05/2005. Date of snowmelt at Calcilarruego, where only dietary plants were sampled, was the 19/05/2005. Degree-days were calculated by summing daily mean temperatures from the date of snowmelt until the plant sampling date. We found a strong positive correlation between sum of degree-days and days from snowmelt (R^2^ = 0.99), and a similar level of correlation between these variables and plant phenophases, which is common in alpine environments [53,54]. Thus, we only used the days after snowmelt for relationships to phenophases, or nutrient changes in Ptarmigan faeces and diet.

### Statistical analysis

To test the effect of phenophase on N content of woody species, we conducted a Student t-test (or Mann-Whitney U-test, when data did not adjust to normal distribution) for two mean comparisons of plant N content between different phenophases. To model the relationship between faecal N and potential N in diet, we performed an Analysis of Covariance (ANCOVA) with potential N in diet as response variable. This analysis aims to predict potential dietary N from the faecal N of a given sample, and allows us to test whether this relationship is independent of year. Generalised Additive Modelling (GAM regression) was used to model the relationships between potential nitrogen in diet and snowmelt time. Since data for the year 2002 was insufficient, tests were restricted to the data for the years 2003 and 2004. Additive modelling fits smoothing curves through the data and is therefore able to describe a wide variety of relationships [55]). GAM was applied here using R-platform and the function gamm of the package mgcv [56]).

Average botanical composition between and within pre- and post-melting periods were compared by means of Spearman rank correlation tests using the 24 most abundant items in the total diet (which comprise the 80% of total identifications). These tests were performed with SPSS and Statistica packages. We utilised Variance Ratio test to check differences between variability of snowmelt and laying dates [57].

## Results

### Diet composition and plant N content

**Fig 1:**
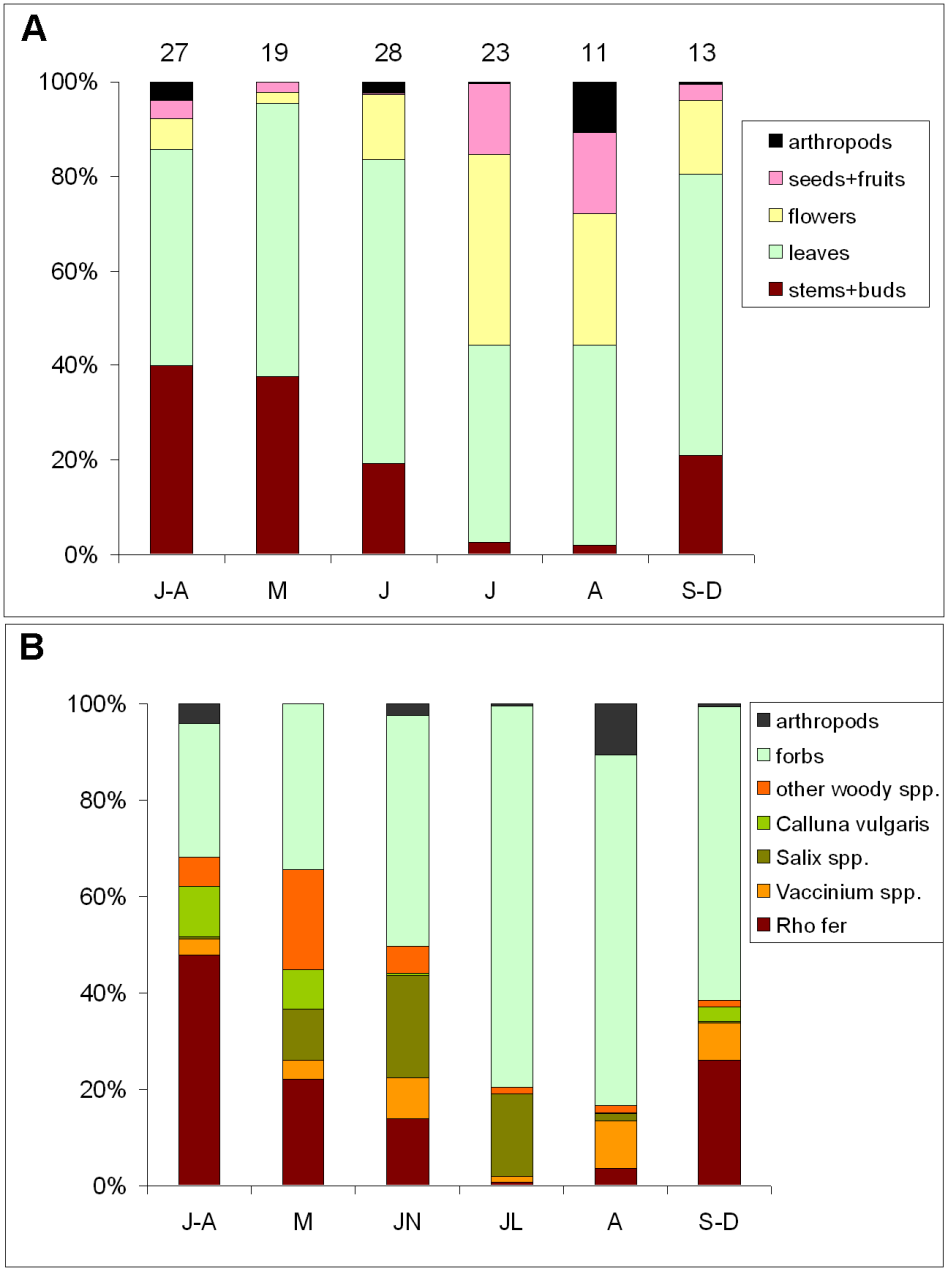
Food types of Rock ptarmigan in Western Pyrenees and their monthly variation. The three samplings years and autumn/winter months have been pooled. Food items are expressed in broad (morphological) (A) and morpho-taxonomical (B) food categories. Numbers above bars indicate sample size.

Nearly half (52%) of the items found in ptarmigan faeces were leaves of dicotyledons (herbaceous and woody) and along with the stems and buds of woody species (22%), they form the bulk of the ptarmigan diet in the Eastern Pyrenees on an annual basis. Flowers (17%), arthropods (3%), and seeds and fruits (6%), which are consumed mainly in summer, completed the rest of the ptarmigan diets over the year (Fig 1). Taxonomically, *Rhododendron ferrugineum* (21%), other Ericaceae (*Calluna vulgaris*, *Vaccinium* spp.) (9%), alpine dwarf willows *(Salix* spp.) (9%), Compositae (*Crepis pyrenaica*, *Leucanthemopsis alpina*, *Leontodon* sp.) (16%) and *Jasione crispa* (5%) were the most abundant species in ptarmigan droppings (Fig 1). Graminoids were nearly negligible (1.7%) in ptarmigan diet, regarding their abundance in alpine pastures.

**Fig 2:**
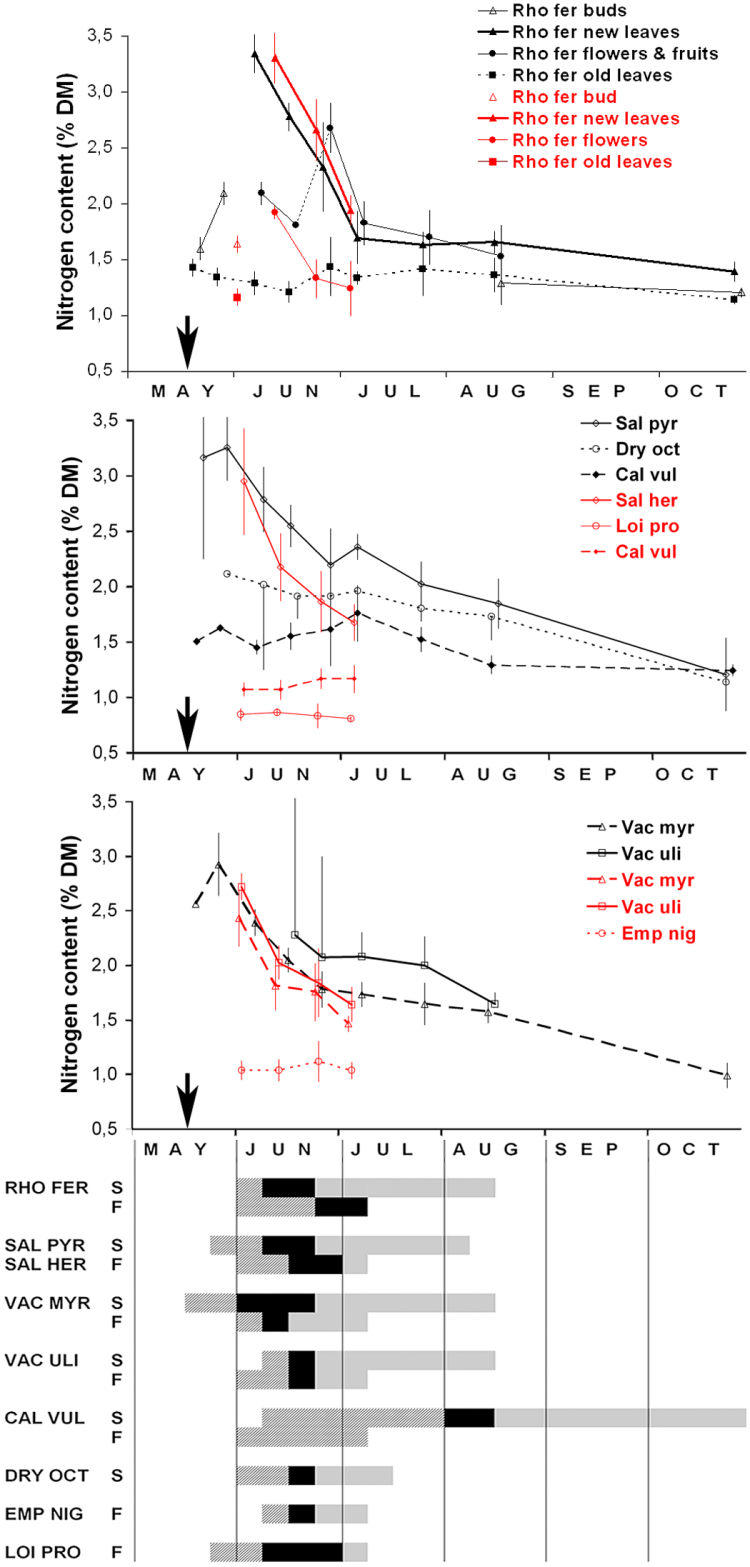
Temporal variation of mean N content (% DM) of the main food species and plant parts of Pyrenean rock ptarmigan in Canigou Massif, France, Eastern Pyrenees (in red) and Calcilarruego, Spain, Middle Pyrenees (in black) during growing and vegetative periods. Species and plant parts are distributed in several figures for clarity reasons. Snowmelt dates are marked by arrows: 16 and 19 May for French and Spanish locations respectively. Plant parts are current year leaves unless otherwise indicated. Vertical bars are 95% confidence limits (only for points with n>3). Below: average phenological stage of sampled species from Spanish (S) and French (F) locations: white, senescence; striped, growing; black, flowering; hatched, fruiting. Species are denoted by the three first letters of genus and species name (see full name in Material and Methods).

The N content variation of the main species in the ptarmigan diet is shown in Fig 2. In Canigou, where the relationship between rock ptarmigan diet and snowmelt was investigated, only results from the reproductive period are represented (Fig 2 in red). The results of the Calcilarruego sampling show a longer temporal extension of N content variation in order to appraise the total annual variation of protein content (Fig 2 in black). The N content variation of the *R*. *ferrugineum* parts (stems, buds, flowers, fruits) is shown in the upper part of Fig 2. Old leaves and current-year leaves of *R*. *ferrugineum* coexisted on the same plant during the growing season. The first had very low and nearly constant N content (1.1%–1.4%). Buds of *R*. *ferrugineum* also have a low N content, but just one week after snowmelt this increased significantly (Fig 2, *t* = 6.4, *p* < 0.001). If buds gave rise to new leaves, the N content again increased considerably ten days later, and was one of the most N-rich dietary item during the growing season, along with *Salix* spp. and *Vaccinium* spp. leaves. If *R*. *ferrugineum* buds gave rise to flowers, the N content was maintained, and then increased when they transformed into immature fruits. During ripening, N content drops. The N content of *Salix* spp and *Vaccinium* spp. leaves peaked earlier than *R*. *ferrugineum*, just seven days after snowmelt, and remained higher over a longer period of time (Fig 2).

The N content depends on the species or plant part, but also on their phenological stage (Fig 2). For woody species in the ptarmigan diet, we found two variation patterns. After snowmelt, most of the species (hump-shaped group) began to grow rapidly and N content increased very quickly, parallel to the sprouting of new leaves (e.g. *R*. *ferrugineum*, *Salix* spp., *Vaccinium* spp.). A few weeks later, N content decreased significantly, more or less suddenly depending on the species (Mann-Whitney U-test: *U* = 321, *p* = 0.02 for *R*. *ferrugineum*; *U* = 40, *p* < 0.001 for *Salix* spp and *U* = 33, *p* < 0.001 for *V*. *myrtillus* between growing and flowering phenophases, respectively). In other species, such as *Empetrum nigrum*, *Dryas octopetala* or *Loiseleuria procumbens* (even group), the N content barely increased during their active period (non-significant differences between growing and flowering phenophases *t* = 0.47 (*p* = 0.65) for *E*. *nigrum*, *t* = 0.56 (*p* = 0.59) for *Dryas octopetala* and *t* = 1.67 (*p* = 0.11) for *L*. *procumbens*). The hump-shaped group of species represented 36% of the total ptarmigan diet, while the even group represented only 6%. Leaves of herbaceous dicotyledons, another important component of the ptarmigan diet (30%), also followed the N time variation pattern of the hump-shaped group (S1 Table).

**Fig 3:**
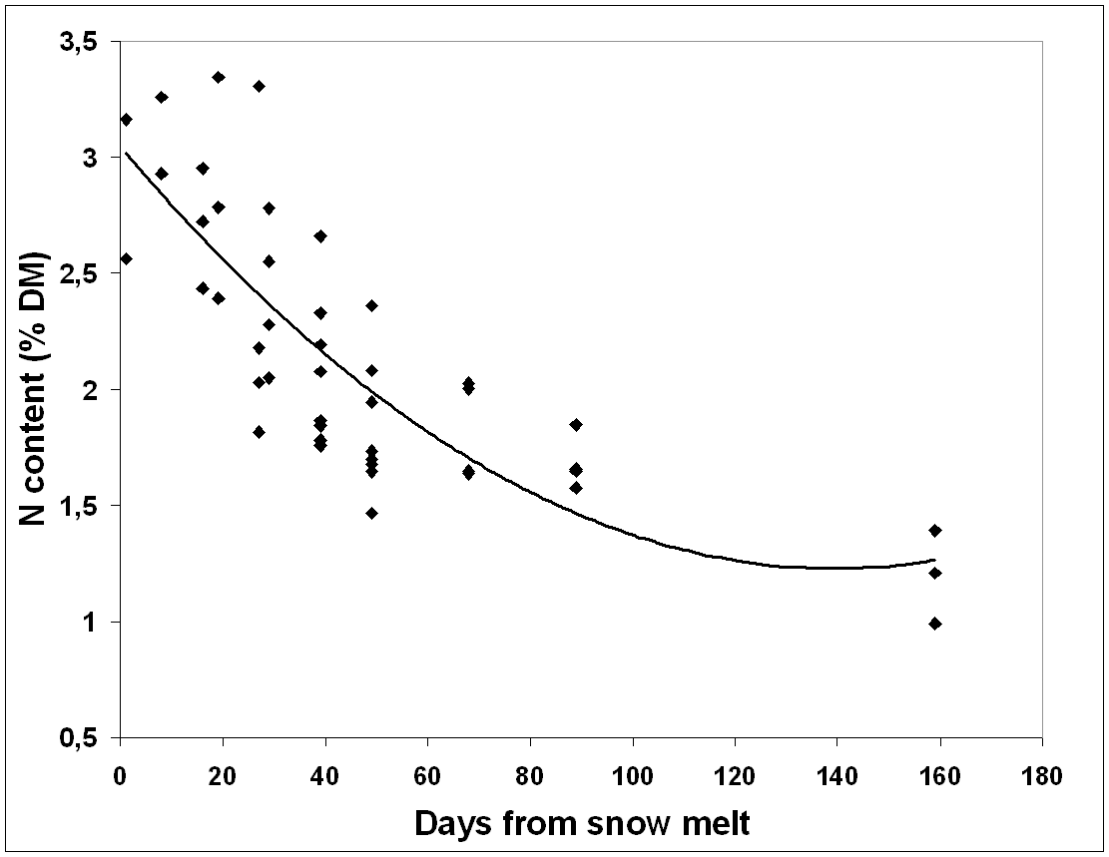
Relationships between days from snowmelt and N content of the main woody species of ptarmigan diet. All species and site samples pooled. Line corresponds to a quadratic adjusted model (see text).

The decline in N content in the shrubs most abundant in the ptarmigan diet (e.g. *R*. *ferrugineum*, *Salix* spp., *Vaccinium* spp.) was related to the number of days after snowmelt (Fig 3). The general model for N decline for the main dietary woody species as a whole during their vegetative period was: N = 0.00009D^2^–0.026D + 3.04 (*R*^2^ = 0.655; d.f. = 45; *p* < 0.001), with N being nitrogen content (% of dry matter) and D the number of days after snowmelt.

### Relationship between free-urate faecal N and potential dietary N

**Fig 4:**
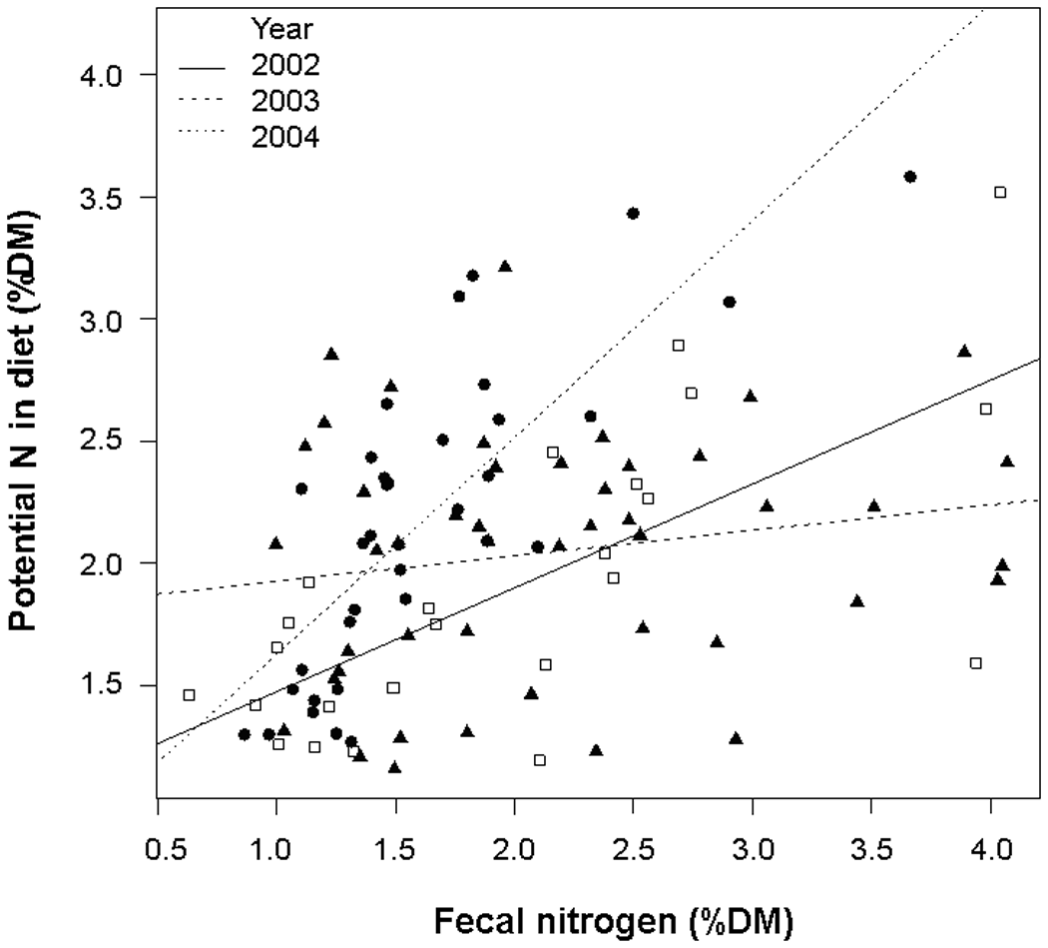
Relationship between potential dietary N and faecal N of Pyrenean Rock Ptarmigan (ANCOVA plot) split by year (2002, 2003 and 2004). Data are expressed in percentage of dry matter (%DM). Empty squares: samples from 2002; black triangles: 2003, and black circles: 2004. See Table 1 for ANCOVA results.

We found a significant relationship between free-urate faecal N and potential dietary N, but this relationship varied depending on the year (Table 1). The ANCOVA explained nearly 40% of total variance (Adjusted *R*^2^ = 0.382). Year 2004 showed the best fitting line (Fig 4). The highest value of N content corresponded to a sample of high arthropod content (i.e. 58%) while the lowest point (0.6%) corresponded to a sample with a high proportion (i.e. 69%) of *Pinus uncinata* leaves (S1 Fig).

**Table 1:** ANCOVA results. Observed and fitted relationship between potential dietary N of Pyrenean Rock Ptarmigan (response variable) and the continuous variable free-urate faecal N (Faecal nitrogen) in the years 2002, 2003 and 2004 (factor Year).

Source	Df	SS	MS	*F*	*p*-value
**Faecal nitrogen**	1	6.12	6.12	30.0	0.0001
**Year**	2	2.72	1.36	6.7	0.002
**Faecal nitrogen×Year**	2	5.27	2.64	12.9	0.0001
**Residuals**	99	20.17	0.20		

Although the fitted relationship between potential nitrogen in diet and faecal nitrogen is positive in all three cases, the slopes vary according to Year. Overall, this model explained Adjusted-*R*^2^ = 0.38 (38%). Df = degrees of freedom; SS = Sum of squares; MS = Mean square.

### Relationship between potential dietary N and snowmelt time

**Fig 5:**
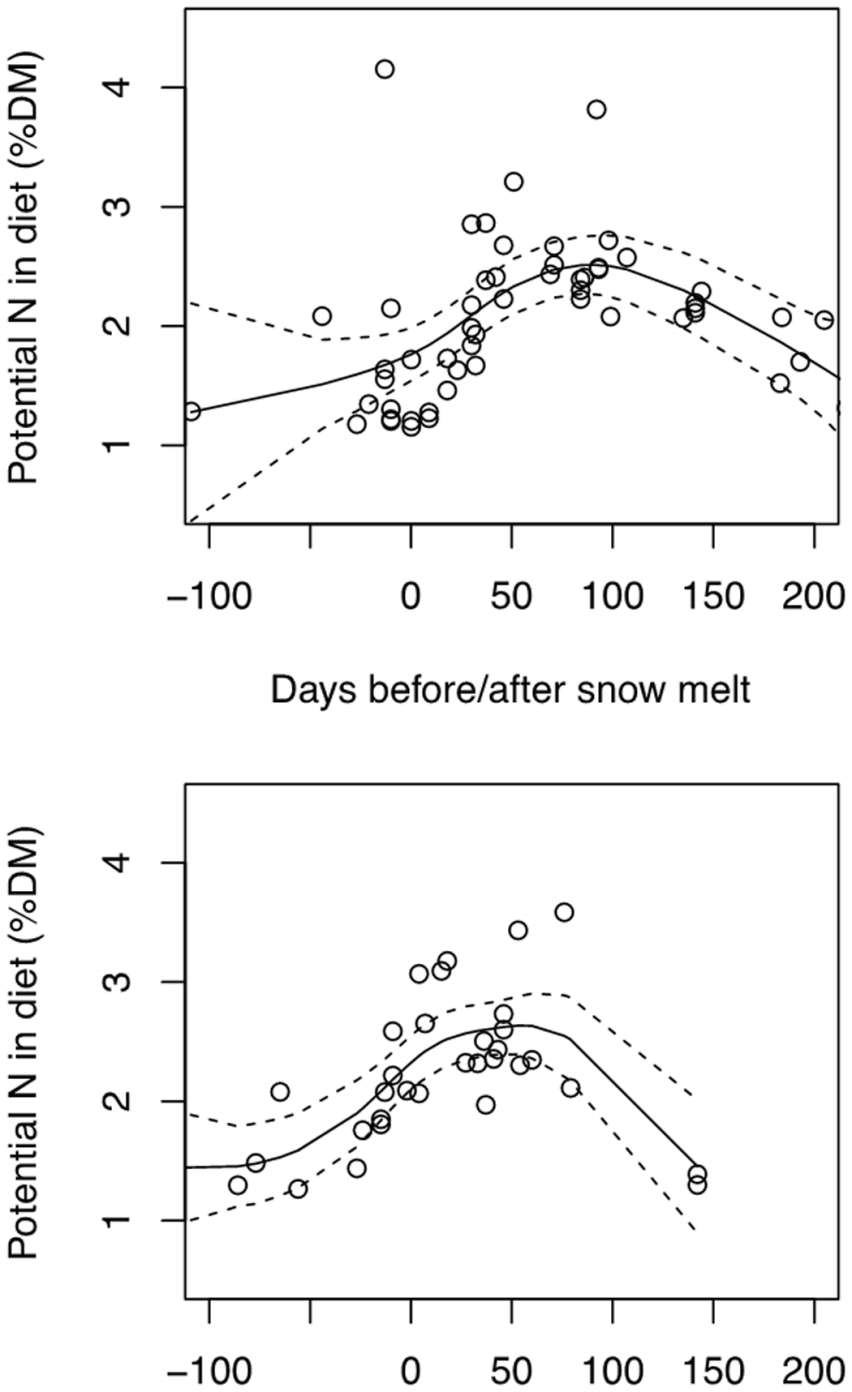
Fitted Generalised Additive Models (GAM plots). The smoothers represent the observed relationship between potential dietary N of Pyrenean Rock Ptarmigan (expressed in percentage of dry matter, %DM) and snowmelt time (indicated in days before/after snowmelt). The upper plot is for 2003 and the lower for 2004. See Table 2 for GAM results.

We rejected in all cases the null hypothesis of no-relationship between potential dietary N and snowmelt time (Table 2). These relationships were hump-shaped and explained *R*^2^ = 0.32–0.57 (32–57% of total variance). Models suggest that the optimum (maximum peak) in nitrogen content occurs at about 50 days after snowmelt (Fig 5).

**Table 2:** Generalised Additive Modelling (GAM) results, where potential dietary N of Pyrenean Rock Ptarmigan recorded in 2003 and 2004 are the response variables and snowmelt time is the predictor.

Response	*n*	adjusted-*R*^2^	*F* (edf)	*p*-value
**Potential dietary N (2003)**	57	0.32	7.48 (3.75)	0.0001
**Potential dietary N (2004)**	35	0.57	11.45 (4.04)	0.0001

Adjusted-*R*^2^ = the variance explained by the model; edf = estimated degrees of freedom; n = sample size.

### Comparison of diet composition before and after snowmelt

The botanical composition of diets before the snowmelt period was not associated (Spearman rank correlation) with botanical composition after the snowmelt period in the three years of the study (Tables 3 and 4). Average diet composition before snowmelt was significantly associated between 2002 and 2003, but neither were associated with that of 2004 before the snowmelt period (Table 3). The diets in 2002 and 2003 during the pre-melting period were characterised by a high proportion of buds, stems and old leaves of *Rhododendron ferrugineum*, all of low nutritional value (Table 3 and S1 Table). The pre-melting period of 2004 was characterised by a high consumption of *Calluna vulgaris* and dicotyledon leaves that at this time had very low nutritive value (Table 3 and S1 Table). The composition of diets after snowmelt in 2002 and 2003 were also correlated, and there were also some similarities to that of 2004 (Table 4). Important quantities of leaves and flowers of herbaceous dicotyledons, *Salix* spp leaves and Asteraceae seeds characterised these types of diets (Table 3).

**Table 3:** Mean values (%) of diet composition of the top 24 diet items (80% of total diet) before snowmelt (from January to the average day of snowmelt) and after snowmelt (from the day of snowmelt to the end of July).

	2002		2003		2004	
Simplified food categories	Before	After	Before	After	Before	After
	*n* = 13	*n* = 16	*n* = 13	*n* = 25	*n* = 15	*n* = 13
*R*. *ferrugineum* buds	15.1	1.7	26.5	2.3	11.6	0.0
*R*. *ferrugineum* stems	9.9	15.2	19.5	7.8	0.4	0.0
*R*. *ferrugineum* leaves	4.7	0.4	5.0	0.0	4.5	0.0
Stem (indeterm.)	7.0	5.4	3.4	2.0	0.5	0.5
*Calluna vulgaris*	4.9	0.0	3.1	0.1	19.9	0.2
*Jasione crispa*	2.7	1.4	2.3	7.9	6.5	3.4
*Salix pyrenaica*	0.5	11.5	0.4	10.6	0.0	0.0
*Salix herbacea*	0.0	13.3	0.2	8.5	0.7	8.5
*Alchemilla catalaunica*	6.0	0.2	0.1	0.7	15.4	0.5
Asteraceae (flowers)	0.0	11.0	0.0	6.0	0.1	17.7
*Crepis pyrenaica*	0.0	9.5	0.0	0.0	0.1	4.2
Forb flower (indet.)	0.1	2.6	0.0	9.4	2.3	5.1
*Oxytropis foucadii*	3.1	2.1	0.0	1.1	7.6	4.7
*Cerastium fontanum*	4.6	2.2	3.6	1.4	0.0	1.4
*R*. *ferrugineum* flowers	2.0	0.1	3.7	0.4	6.1	0.0
Forb leaf (indeterm)	0.1	0.9	0.2	0.8	2.1	8.4
Arthropods	0.6	1.2	6.7	0.8	1.2	1.4
Campanulaceae	0.0	0.2	0.0	6.0	0.0	5.6
*Leontodon* sp. seed	0.0	1.8	0.0	4.8	0.0	4.1
*Anthyllis vulneraria*	10.6	0.0	0.0	0.0	0.0	0.0
Asteraceae seed	0.0	3.5	0.0	0.2	0.4	5.0
*Hieracium lactucella*	0.2	0.0	4.6	0.1	2.9	1.3
*Pinus uncinata*	6.8	0.0	1.5	0.0	0.5	0.0
*Vaccinium myrtillus* stem	5.8	0.0	0.4	0.6	0.7	0.0

Diet items are leaves unless otherwise indicated.

**Table 4:** Values of Spearman correlations (Rho) between the top 24 diet items (Table 3) of the ptarmigan diet before and after snowmelt.

	2002 after	2003 before	2003 after	2004 before	2004 after
**2002 before**		0.45*			-0.76**
**2002 after**	-		0.64**		0.39^a^
**2003 before**	-	-			-0.57**
**2003 after**	-	-	-		
**2004 before**	-	-	-	-	

Diet items are leaves unless otherwise indicated.

Only significant results are shown.

* *p* < 0.05;

** *p* < 0.01;

^a^ *p* = 0.056.

## Discussion

### Diet composition and plant N content

After spring snowmelt, alpine plants usually experience a rapid growth, which is normally associated with a rise in nutrients (mainly protein content) and an increase in digestibility [58–60]. Plant phenophases and subsequent nutrient variation are generally linked to the increase in temperatures or days from snowmelt, and are species-specific [52,61,62]. *Rhododendron ferrugineum* along with *Salix* spp. and *Vaccinium myrtyllus* are some of the most abundant components of the rock ptarmigan diet in the Pyrenees (Fig 1, [63,64]) as well as in some areas of the Alps [44]. An important N increase in the new leaves of *Rhododendron ferrugineum* occurs 20–25 days after snowmelt. However, in *Salix* spp. and *Vaccinium myrtilus* the peak of N content is only seven days after snowmelt and 15 days earlier than in *Rhododendron ferrugineum* (Fig 2). This also coincides with the sprouting sequence found for the same species in the Alps [52]. Theurillat & Schlüssel [52] found that the sum of degree-days for bud opening in *Vaccinium myrtilus* is -14° ± 24° (beginning before the snow thaw), while for *Salix herbacea* it is 11° (no variability was given), and 194° ± 20° for *Rhododendron ferrugineum*. The concentration of N drops soon after the end of the growth phase and when flowering begins (Fig 2). Adjusting the timing of laying to match the peak of N content is important for reproductive success in birds [2,9] and protein content is a principal component of food quality [45,46]. To use the maximum protein content of the dietary plants for the benefit of reproduction, ptarmigan females should not significantly delay the timing of laying after the snowmelt. The high N content of the dietary plants of ptarmigan lasts barely a month before declining sharply (Fig 2).

### Date of snowmelt, diet quality and timing of breeding

ANCOVA analysis suggests that free-urate faecal N is a good estimator of dietary N content and hence of the digestible protein consumed by ptarmigan, but variance explained was quite low and the estimation is not independent of year sampled. Thus we recommend being cautious when using this variable as dietary N estimator. Potential dietary N shows a hump-shaped pattern of temporal variation throughout the years (Fig 5), with a maximum peak coinciding with the increase in diet quality during the breeding season and minimum values coinciding with winter shortages. The analysis of nutrient content of rock ptarmigan crops in Alaska provided a similar pattern of seasonal variation [65]. The rise in N content in the ptarmigan diet is linked to the increase in N content of plants consumed, and this depends on the timing of snowmelt and temperatures rising [52]. Until plants begin to grow, they do not seem to have sufficient N content to allow the breeding onset of ptarmigan hens. According to Moss [66], domestic hens need more than 2.8% of N content in the diet to breed (see also [8]). A delay in the growth of key dietary species may negatively affect the nutrition level of ptarmigan females at the beginning of the breeding season, and beyond their reproductive success [12,25,26]. In willow grouse (*Lagopus lagupus*), females produce fewer young when they are in poor physical condition [67].

Diet quality is influenced by the type of food consumed [30] but also by phenological stage. Although botanical diet composition before snowmelt is different from that of the post-melting period [44] and varied between years (Table 4), potential dietary N content did not differ significantly. Until snowmelt, diet quality is very low. Dietary N contents rise significantly after snowmelt, regardless of species or items consumed (Fig 5). What seems to matter for food quality is the N content of dietary plants, less than the species consumed, although there are species such as *Calluna vulgaris*, *Empetrum nigrum* or *Loiseleuria procumbens* that always present a low N content ([68] and Fig 2). In fact, the consumption of some species such as *Rhododendron ferrugineum* or *Calluna vulgaris* on an annual basis is inversely correlated to N content in the Pyrenean ptarmigan diet [30].

Two important variables determining reproductive success are the age of breeding birds and the availability of food, the latter being an important determinant (among other factors) of the quality of the habitat [69–71]. In general, older individuals nest earlier, produce larger clutches and have greater breeding success [69,72,73]. Late laying dates are often associated with a low reproductive success. For example, in Barnacle goose (*Branta leucopsis*), a delay in the incubation period due to a late snowmelt negatively affected the proportion of pairs starting to breed, the nest success and the brood size at hatch [9]. Late springs can reduce the size and number of eggs, or the chick weight at birth of White-tailed ptarmigan (*Lagopus leucura*), decreasing their probability of survival [6]. Chicks that are born later in the breeding season do not have enough time to develop or to build fat reserves for the winter, and their mortality probabilities increase [7]. The studied population of Pyrenean ptarmigan revealed a significant inverse relationship between the date of snowmelt and reproductive success defined as the ratio of young to adult in August [15].

**Fig 6:**
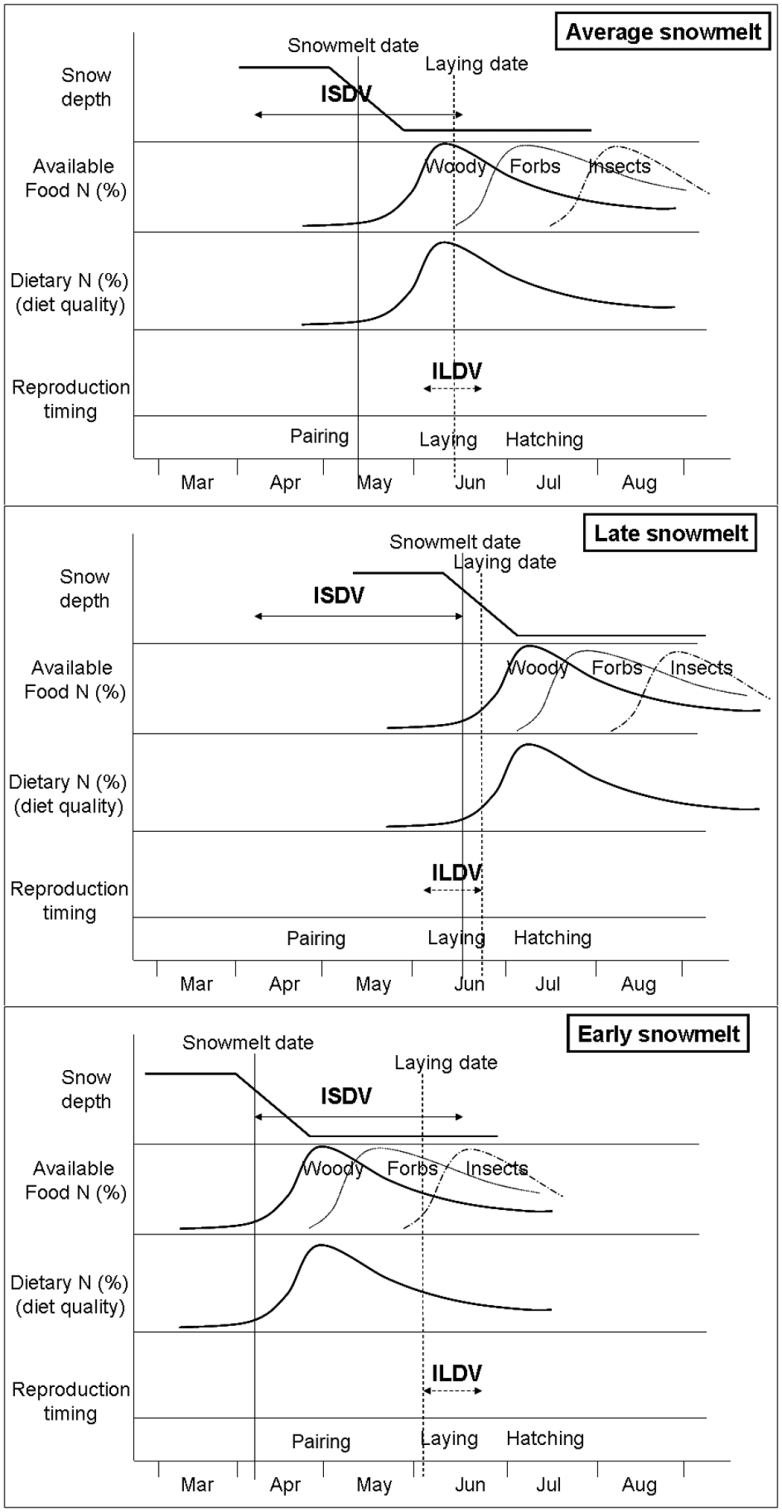
Explanatory model showing possible relationships between date of snowmelt, diet quality and reproductive success in Pyrenean ptarmigan. Average and both extremes of interannual snowmelt variation are shown. Snowmelt determines the beginning of growth and N content in the plants fed on by ptarmigan, which in turn regulates the N content of hen diets and protein increases during reproduction period. Previous data (Novoa et al. 2008) show that interannual variability of laying date (ILDV) are significantly less than interannual variability of snowmelt date (ISDV). Thus, in years of late snowmelt hens have less time to obtain good-quality food, resulting in poor body condition for laying and incubation, and diminishing breeding success. In years of early snowmelt, there is no loss of protein gain for ptarmigan hens, because of the asymmetric size of N content curve in plants and the appearance of new N peaks of major summer foods for ptarmigan (forbs and insects).

Evolutionarily, it is accepted that birds try to match laying dates with the maximum abundance of food resources [1,26]. In arctic-alpine environments, these maximum values of energy and nutrients are associated with the timing of snowmelt [28,58,59], but this is normally subject to a high temporal variability [54]. When the date of snowmelt is delayed (late springs), Pyrenean ptarmigan females also delay the timing of laying [15]. If they do not, laying coincides with the beginning of an ascending phase of the plant nutrient cycle with low nutrient values (Fig 6 middle), resulting in the malnutrition of hens. Nevertheless, the range of variation of laying date seems to be narrower than that of snowmelt (ILDV < ISDV, Fig 6) and this imposes a limit to the delay in laying. From the data collected by Novoa et al. [15], it can be inferred that an inverse relationship exists between the date of snowmelt and the interval between snowmelt and hatching date from 1997 to 2006, in the same studied population (*R*^2^ = 0.84, *p* < 0.01, *n* = 10). Thus, as snowmelt is delayed, the interval between snowmelt and hatching is reduced (and hence laying [74]). In late springs, ptarmigan hens could be "forced" to lay without having reached an optimal level of protein intake.

Conversely, early spring (Fig 6 below) normally leads to a higher reproductive success [15], probably as a result of improved nutrition in females [2, 25]. It could be argued that, as in the previous case, an advance of the spring could also produce a mismatch between the peak abundance of plant nutrients and laying date [75,76]. If laying coincides with the decline of the hump-shaped curve (Fig 4), a malnutrition of females could take place, lowering reproductive success [7]. The main difference with late snowmelt is that the sinusoidal curve of nutrient plant variation is leftward skewed, with the increase being very fast and the decline in N content very slow (Fig 4). Several studies in arctic-alpine environments confirm this pattern of temporal variation of N content and other nutrients in plants [58,77,78]. In addition, once the peak of highest N content in the woody species of the ptarmigan diet has been surpassed (continuous line of second row in Fig 6), peaks of other basic foods in the ptarmigan diet successively appear, i.e. dicotyledonous forbs and insects, which have later abundance peaks. The proportion of forbs and arthropods in the ptarmigan diet grew in July and August, respectively [30], coinciding with their greater abundance in Pyrenean alpine habitats [34,79]. In this way, the protein provision in the right side of the sinusoidal curve is ensured. Thus, females that lay early obtain a better nutrient supply and in general increase their chances of reproductive success [25].

The interannual variability of laying date (ILDV) seems to be significantly smaller than the interannual variability of snowmelt date (ISDV) (Fig 6). In the case of the Pyrenean ptarmigan, the date of snowmelt varied by 79 days in Canigou between 1997 and 2006, whereas the median date of laying varied by only 30 days [15]. Snowmelt variation was 63 days in the Calcilarruego sampling area between 1993 and 2012 [54]. The date of snowmelt (linked to the snow pack), tends to experience large annual fluctuations associated with the North Atlantic Oscillation index [80]. In the Mountain White-crowned Sparrow (*Zonotrichia leucophrys*) [81], the date of 25% snow cover varied by 48 days in four years, while the average date of laying ranged only 17 days. The interannual variability of the average date of laying in other species of grouse was also quite reduced, i.e. 16 days in 8 years for *Lagopus lagopus* [7] and 17 days in 16 years for *Dendragapus canadiensis* [82]. In the ptarmigan population studied, the variance of the snowmelt date was significantly greater than that of laying (ISDV > ILDV in Fig 6; test of homogeneity of variance *F* = 5.3, *p* < 0.01) and this supports the initial hypothesis of the relationship between snowmelt and reproductive success mediated by food quality.

### Climate change and breeding success in Pyrenean ptarmigan

The Pyrenean ptarmigan occupies one of the southernmost ranges of the species worldwide distribution. It has been suggested that populations near the limit of range distribution may be more vulnerable to the effects of climate change [83,84]. One of these effects is that the alpine species adapted to a cold environment may be faced with an unfavourable habitat if weather becomes warmer. In fact, some models predict the disappearance of Pyrenean ptarmigan in a future scenario of temperature increases [85,86]. Nevertheless, the increase in temperatures could also have a favourable effect on the reproductive success of the Pyrenean ptarmigan. If temperatures rise there could be an advance of plant growing season, improved nutrition level of hens, and a higher reproductive success, providing Pyrenean ptarmigan have the adaptive flexibility to sufficiently advance their timing of laying (i.e., phenotypic plasticity, [3,87]).

Various studies demonstrate an advance in the phenology of plants and animals as a result of climate change [3,88–90]. In some migratory insectivorous birds (e.g. European pied flycatcher [*Ficedula hypoleuca*]) an advancement of the peak abundance of their prey (caterpillars) in relation to laying date, caused a decrease in their reproductive success [5,91]. In the case of the Pyrenean ptarmigan the effect could be the opposite. First, the basic foods of herbivorous birds are not subject to such narrow pulses of abundance as that of insectivorous birds, occurring more gradually over time (Fig 6). In addition, because of the nature of the nutrient availability curve, the low level of resources is not as important on the right side of the curve (early snowmelt), as on the left side (late snowmelt).

A decline in snow pack and an advance in snowmelt date have been observed over the last 50 years in the Pyrenees [80]. According to the predictions of recent models on climate change, winter precipitations may decrease in the Pyrenees over the course of this century, with a greater incidence in the Eastern sector [92]. However, there are still no conclusive studies of how this will affect the reproductive performance in birds [89,93]. Although earlier laying of clutches is often associated with larger clutch sizes and greater production of young, the effects of earlier laying on reproductive performance (i.e. population dynamics, recruitment) are less clear [3].

In temperate ecosystems, temperature increase tends to have a direct influence on the start of the growing season [94]. In alpine environments the advancement or delay of the plant growing season depends on the disappearance of snow cover [52,95], which in turn depends on temperature, but also on other factors. High altitude, northern exposure or late spring snowfall can cause the presence of a thick snowpack and a delay in the snowmelt in alpine environments [80]. Variability and unpredictability of rainfall in alpine ecosystems are higher than in areas of low altitude [15,96] and climate change may increase this variability [97]. As we have previously shown, the date of laying could depend on less variable factors (such as photoperiod) than climate variability [98]. As in other birds, the impact of climate change on Pyrenean ptarmigan reproduction may depend on their adaptive capacity to adjust their timing of laying [87].

In this study, we found that free-urate faecal N content was related to the protein content of the ptarmigan diet. Therefore, the number of breeding periods analysed (only three years) is insufficient to find conclusive relationships between the N content of the ptarmigan diet during the post-melting period and their reproductive success. We need to further investigate the relationships between diet, plant phenology, weather and reproductive success of ptarmigan to consolidate the assumptions proposed in this work. An individual survey of diet quality of females during the breeding season related to reproductive success would likely be required. The understanding of such relationships will allow the development of models that predict the potential impact of climate change on the population dynamics of the Pyrenean rock ptarmigan and other alpine birds.

## Supporting Information

S1 Fig Distribution of free-urate faecal N (circles) and potential dietary N estimated from microhistological composition (crosses) throughout sampling period (2002–2004). Each point corresponds to a single faecal sample. Arrows indicate the date of snowmelt at nearest weather station.
(TIF)

S1 Table Mean values of N content (% of dry matter) of different food items utilised to estimate the potential N content of each individual diet determined by microhistological analysis. Winter period comprises from September to date of snowmelt. Diet items are leaves unless otherwise indicated.
(PDF)
