# Supplementary figures and images for: Influence of Snowmelt Timing on the Diet Quality of Pyrenean Rock Ptarmigan (Lagopus muta pyrenaica): Implications for Reproductive Success
Source: PLoS One. 2016 Feb 5;11(2):e0148632. doi: 10.1371/journal.pone.0148632 (PMC4746074; doi:10.1371/journal.pone.0148632)

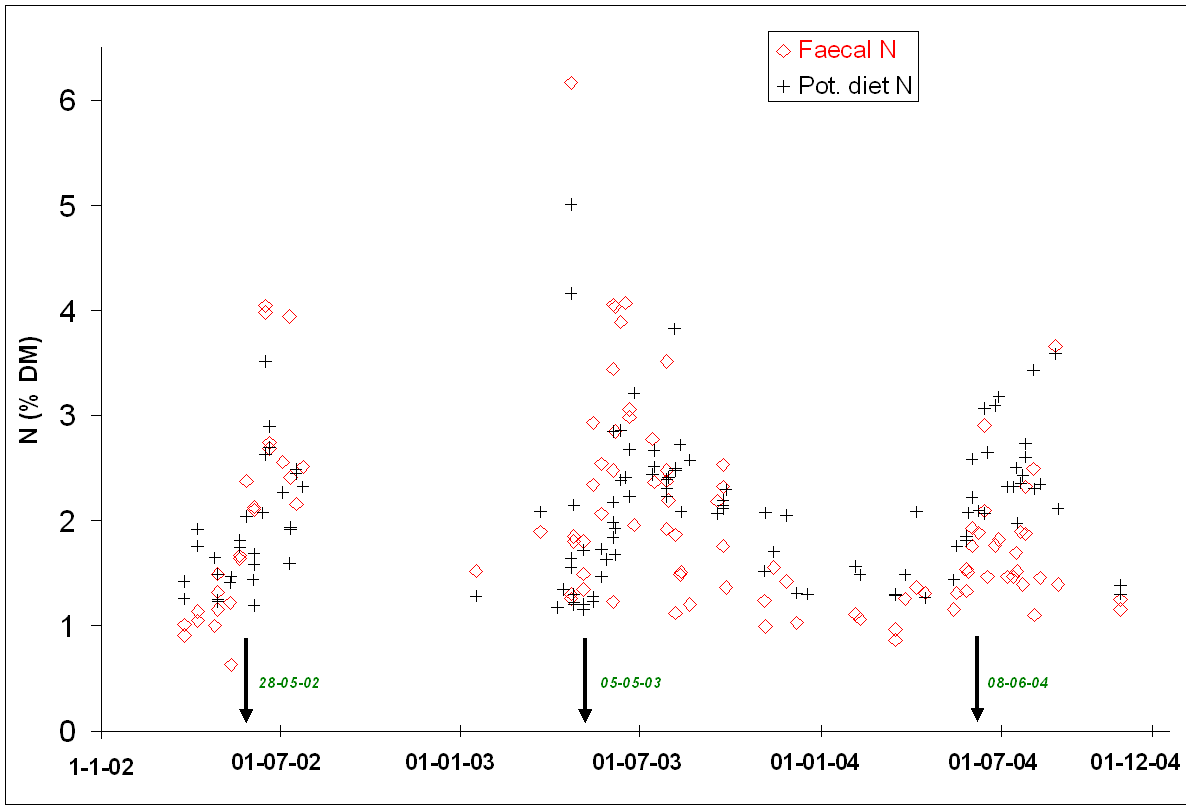

Supplement: S1 Fig — Each point corresponds to a single faecal sample. Arrows indicate the date of snowmelt at nearest weather station. (TIF) [file pone.0148632.s001.tif]
