# Supplementary material for: Influence of Snowmelt Timing on the Diet Quality of Pyrenean Rock Ptarmigan (Lagopus muta pyrenaica): Implications for Reproductive Success
Source: PLoS One. 2016 Feb 5;11(2):e0148632. doi: 10.1371/journal.pone.0148632 (PMC4746074; doi:10.1371/journal.pone.0148632)
Supplement: S1 Table — Winter period comprises from September to date of snowmelt. Diet items are leaves unless otherwise indicated. (PDF) [file pone.0148632.s002.pdf]

S1 Table. Mean values of N content (% of dry matter) of different food items utilised to estimate the potential N content of each individual diet determined by microhistological analysis. Winter period comprises from September to date of snowmelt. Diet items are leaves unless otherwise indicated.

Date	May	June	July	August	Winter	Reference
Arthropod	7.4	7.4	7.4	7.4	7.4	Klasing (1998)
R. ferrugineum flowers	2.0	2.0	1.6	1.6	1.6	this study
Gram ears (mean 7 spp.)	1.6	1.6	1.6	1.6	1.6	Marinas & Garcia-Gonzalez (2006)
Forb flowers (mean 5 spp.)	2.2	2.2	2.2	2.2	2.2	Marinas & Garcia-Gonzalez (2006)
R. ferrugineum bud	2.0	1.6	1.5	1.3	1.2	this study
R. ferrugineum stem	1.1	1.1	1.1	1.1	1.1	this study
R. ferrugineum spp. (stem+bud)	1.2	1.2	1.2	1.2	1.2	this study
R. ferrugineum (stem+bud)	1.0	1.0	1.0	1.0	1.0	this study
Rest of species (stem+bud)	1.0	1.0	1.0	1.0	1.0	this study
Graminoid (mean 13 spp.)	0.9	2.3	1.4	1.3	0.9	Marinas & Garcia-Gonzalez (2006)
Dicot forb (mean 10 spp.)	2.1	3.3	2.4	2.1	2.1	Marinas & Garcia-Gonzalez (2006)
Calluna vulgaris	1.6	1.3	1.4	1.3	1.2	this study
Dryas octopetala	2.1	1.9	1.9	1.7	1.1	this study
Loiseleuria procumbens	0.8	0.9	0.8	0.8	0.8	this study
Pinus uncinata	1.2	1.2	1.7	1.4	1.2	Garin et al (1996)
Rhododendron ferrugineum	1.4	2.8	1.7	1.7	1.1	this study
Salix herbacea	1.7	2.3	1.7	1.7	1.7	this study
Salix pyrenaica	3.2	2.5	2.2	1.8	1.2	this study
Salix spp.	3.2	2.4	1.9	1.8	1.4	this study
Dryas/ R. ferrugineum	1.8	2.4	1.8	1.7	1.1	this study
Dryas/Salix	2.7	2.2	1.9	1.8	1.3	this study
Vaccinium myrtillus	2.9	2.1	1.6	1.6	1.0	this study
Vaccinium uliginosum	1.6	2.2	1.9	1.6	1.6	this study
Vaccinium spp.	1.3	2.1	1.8	1.6	1.3	this study
Polygonum viviparum (bulbils)	2.8	2.9	2.8	2.8	2.8	Moss (1968); Moss & Parkinson (1975)
R. ferrugineum fruit				1.5	1.5	this study
Vaccinium spp. fruit					1.3	Filacorda et al. (1997); Moss & Parkinson (1975)
Asteraceae seed			3.4	3.4	3.4	Fenner (1986)
Seed (indeter.)			3.4	3.4	3.4	Fenner (1986)
graminoid seed			2	2	2	Klasing (1998)

Klasing KC. Comparative Avian Nutrition. Oxon: CAB International; 1998.

Marinas M, García González R. Preliminary data on nutritional assessment of abundant species in alpine pastures of the Pyrenees. Pirineos. A Journal of Mountain Ecology 2006; 161: 85-109.

Garin I, Azorín J, Aldezábal A, García-González R. Implicaciones nutritivas del contenido en taninos de varias especies leñosas. In: XXXVI Reunión Científica de la SEEP; 1996; La

Rioja: Gobierno de La Rioja. Consejería de Agricultura, Ganadería y Desarrollo Rural; 1996. p. 293-298.

Moss R. Food selection and nutrition in Ptarmigan (*Lagopus mutus*). In: Crawford MA, editor. Comparative Nutrition of Wild Animals. London & New York: Academic Press; 1968. p. 207-216.

Moss R, Parkinson JA. The digestion of bulbils (*Polygonum viviparum* L.) and berries (*Vaccinium myrtillus* L. and *Empetrum* sp.) by captive ptarmigan (*Lagopus mutus*). British Journal of Nutrition 1975; 33(2): 197-206.

Filacorda S, Sepulcri A, Piasentier E, de Franceschi PF. Estimation of the chemical composition of black grouse Tetrao tetrix diets in the eastern Italian Alps. Wildlife Biology 1997; 3: 187-
